# Supplementary material for: Mapping the architecture of protein complexes in Arabidopsis using cross-linking mass spectrometry
Source: Proc Natl Acad Sci U S A. 2026 Aug 12;123(33):e2519615123. doi: 10.1073/pnas.2519615123 (PMC13486740; doi:10.1073/pnas.2519615123)
Supplement: Supplementary file 1 [file pnas.2519615123.sapp.pdf]

PNAS

Supporting Information for

Mapping the Architecture of Protein Complexes in Arabidopsis Using Cross-Linking Mass Spectrometry

Cao Son Trinh, Ruben Shrestha, Pengzhi Mao, William C Conner, Andres V Reyes, Sumudu S Karunadasa, Annie Yu, Grace Liu, Ken Hu, Shou-Ling Xu

corresponding author: Shou-Ling Xu
Email: Shouling.Xu@ucsf.edu

This PDF file includes:

Supporting text
Figures S1 to S18
Legends for Datasets S1 to S5
SI References

Other supporting materials for this manuscript include the following:

Datasets S1 to S5

Supporting Text

Extended Materials and Methods

Plant growth condition

Arabidopsis thaliana wild-type (Col-0) seeds were sterilized and sown on ½ Murashige and Skoog (MS) agar square plates supplemented with 0.6% (w/v) phytoblend and lined with nylon fabric. After stratification at 4°C for 3 days, the plates were transferred to growth conditions.

Light-grown seedlings: plants were vertically grown in a growth chamber at 22°C under a 16-hour light / 8-hour dark cycle for 10-12 days, then flash frozen in liquid nitrogen and stored at -80°C for crosslinking experiments on cell lysates and isolated chloroplasts. Dark-grown seedlings: plants were grown horizontally at 22°C in complete darkness for 3 days in a growth chamber, then flash frozen in liquid nitrogen and stored at -80°C for nucleus isolation. Dark growth was used to reduce Rubisco content.

Frozen Arabidopsis tissue was cryo-ground to a fine powder. Different amounts of tissue were used for cross-linking experiments: 6 g from 10 d seedlings for cell lysates, 195 g from 12d seedlings for chloroplasts, and 32 g (replicate 1 with PhoX) or 14 g (replicate 2 with tbu-PhoX) from 3d dark-grown seedlings for nuclei.

diGly proteomics analysis to identify ubiquitinated proteins *in vivo* and datamining

Metabolically labeled Arabidopsis plants with ^{14}N and ^{15}N isotope were harvested, followed by protein extraction and trypsin digestion as previously described (1, 2). Ubiquitinated peptides were enriched using the PTMScan® Ubiquitin Remnant Motif (K-ε-GG) Kit (Cell Signaling Technology) according to the manufacturer's instructions. The enriched peptides were then analyzed by LC-MS/MS. Peptides identified in both light and heavy forms were considered high confidence. Ubiquitination sites were further identified by data mining as described in (3).

Subcellular localization analysis of proteins using SUBA5

The subcellular localization of cross-linked proteins was analyzed using SUBA5 (https://suba.live/). Accession IDs of proteins identified in both intra- and inter-protein cross-links were submitted as input.

Only the localization consensus data were used for classification. Proteins assigned to multiple compartments were counted once in each corresponding localization category when calculating total counts.

PANTHER protein class analysis and mapping intralinks to PDB and AlphaFold structures using ComMap

Arabidopsis gene identifiers (ATG accession numbers) were used for protein class analysis using PANTHER™ Protein Class tool (4), which contains 210 classification terms (version 19.0, released 2024-06-20).

To map the results of intralinks against experimentally determined structures from the Protein Data Bank (PDB) and predicted structures from AlphaFold protein structure database, the protein accession IDs were first converted to UniProt IDs. The intralink information was then mapped to available structures using ComMap, a tool that enables large-scale structure-based mapping by integrating XL-MS data with existing structural models and performing distance calculations (5).

Histone-TurboID Proximity-labeling

Histone H2B (H2B6, AT3G45980) was cloned into the Gateway system and recombined into the destination vector pB7m34GW to generate the ACINUSpro::H2B6-TurboID-VENUS construct, which was introduced into *Arabidopsis* wild-type plants via floral dipping. T2 transgenic seedlings were used for TurboID experiments. Plants expressing ACINUSpro::TurboID-YFP-NLS served as controls. TurboID sample preparation, data acquisition, and analysis were performed as previously described (6, 7).

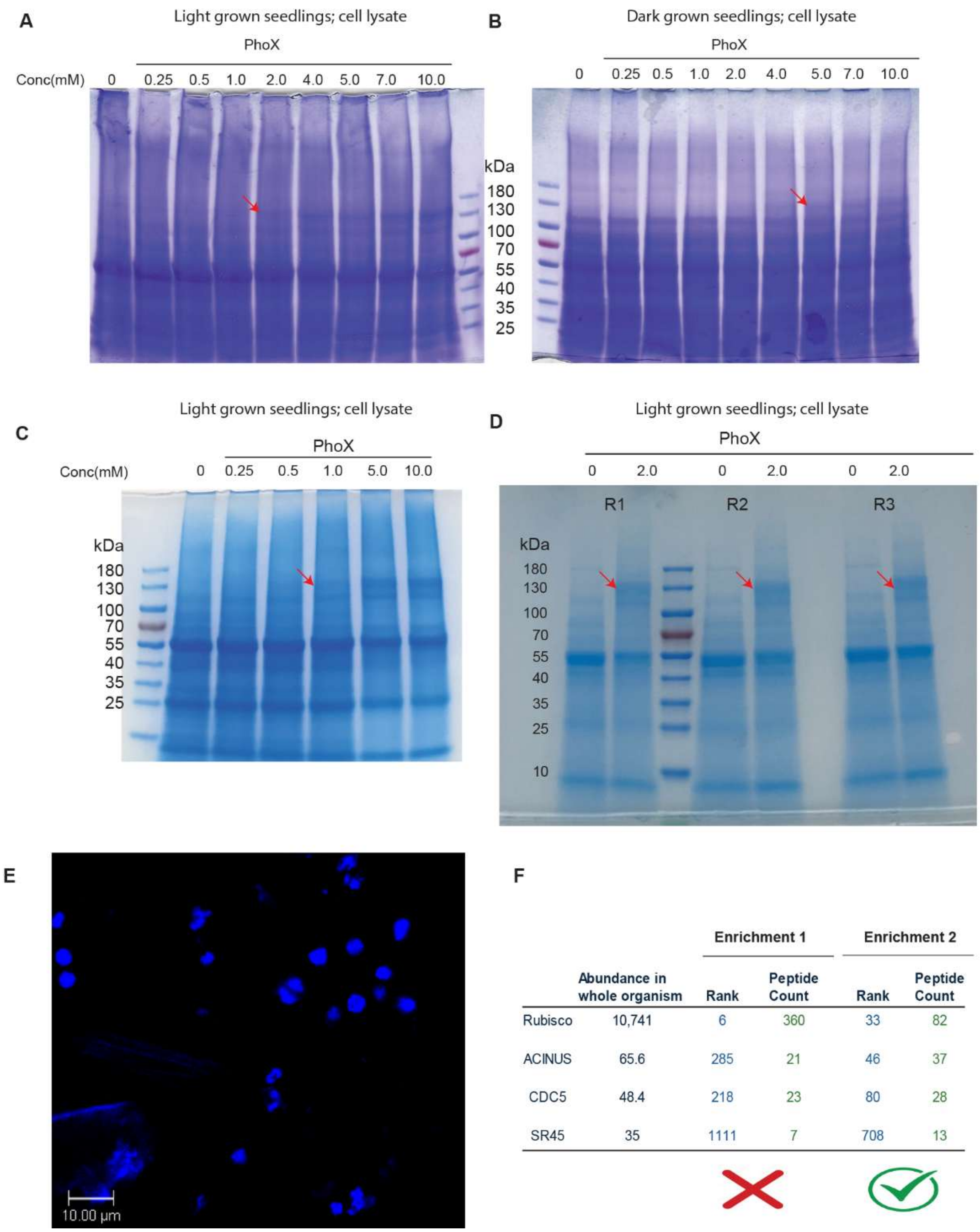

		Enrichment 1		Enrichment 2	
	Abundance in whole organism	Rank	Peptide Count	Rank	Peptide Count
Rubisco	10,741	6	360	33	82
ACINUS	65.6	285	21	46	37
CDC5	48.4	218	23	80	28
SR45	35	1111	7	708	13

Supplementary Fig. S1: Optimization of the PhoX cross-linker concentration for XL-MS. (**A-D**) Different concentrations of PhoX cross-linker (0 to 10 mM) were tested on cell lysates from light (A, C, D) or dark grown seedlings (B). Increased protein cross-linking, indicated by the accumulation of higher molecular weight proteins (red arrow), was observed with increasing cross-linker concentration. However, no additional visible cross-linking was detected at higher concentrations (7-10 mM). (**E**) Representative DAPI-stained image of isolated nuclei. Scale bar = 10 µm. **(F)** Evaluation of nuclear enrichment efficiency. Four protein markers were assessed based on spectral peptide counts and abundance ranks, with whole-organism protein abundance obtained from PaxDB. Improved nuclear enrichment is reflected by increased

spectral peptide counts and higher abundance rankings (i.e., lower rank numbers) for nuclear marker proteins (ACINUS, CDC5, and SR45), and by the opposite trend for the contaminant protein Rubisco.

A

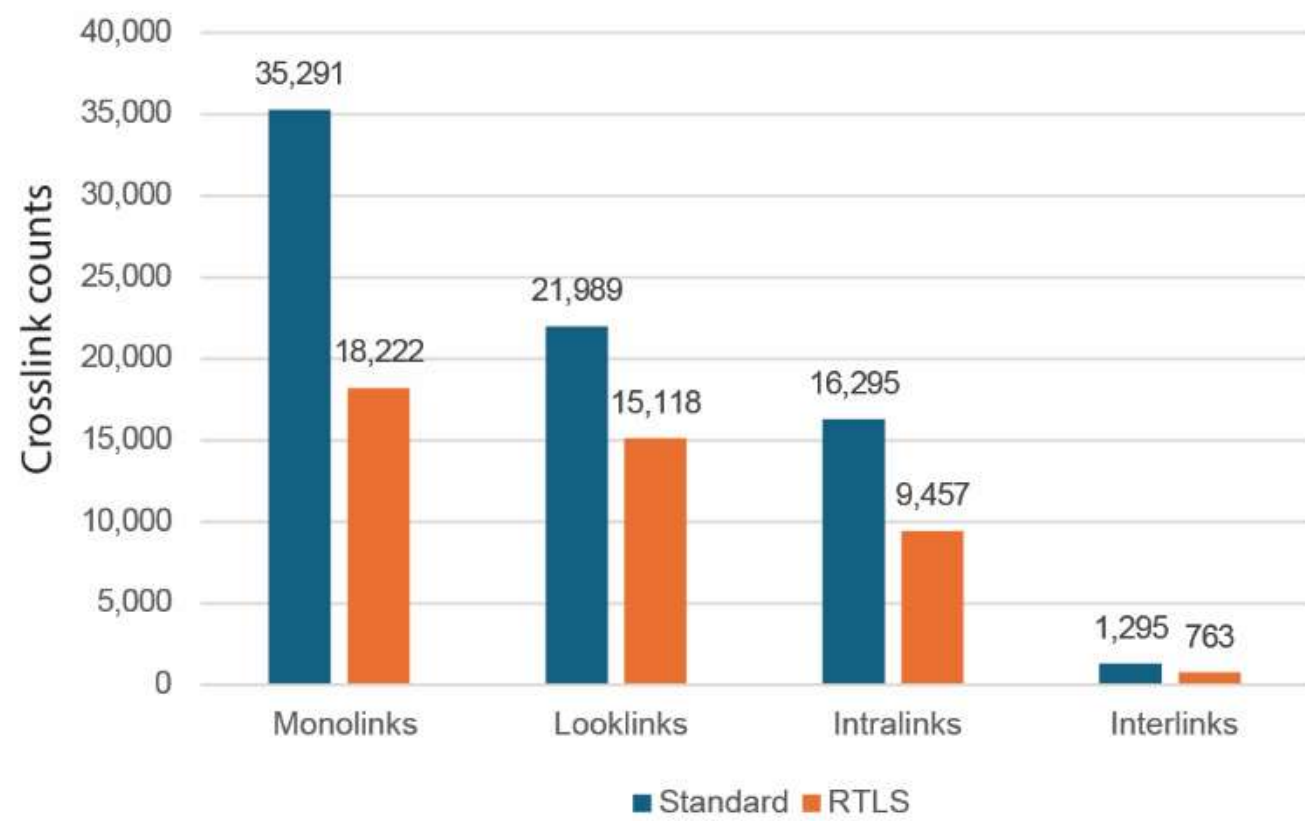

B

	LysateXL		ChloroplastXL		NucleusXL	
	Number	%	Number	%	Number	%
Cross-Linked Peptides	16,486	21.5	18,780	16.6	24,478	18.3
Loop-Linked Peptides	19,233	25.0	21,476	18.9	31,897	23.9
Mono-Linked Peptides	30,946	40.3	54,823	48.4	50,845	38.1
Phospho peptides	3,157	4.1	5,636	5.0	10,962	8.2
Regular peptides	6,955	9.1	12,641	11.1	15,208	11.4

C Entrapment-estimated FDP calculation

FDR control	1% at peptide pair level					
Dataset	LysateXL		ChloroXL		NucleusXL	
Entrapment Database	Lysate11734_Human11734KR-xl0		Chloro9798_Human9798KR-xl0		Nucleus14643_Human14643KR-xl0	
FDP report level	Peptide pairs	PPIs	Peptide pairs	PPIs	Peptide pairs	PPIs
Total FDP (%)	0.18	0.78	0.12	0.58	0.25	1.36
Intra-link FDP (%)	0.00	0.00	0.00	0.00	0.00	0.00
**Inter-link FDP (%)**	2.76	**4.47**	0.42	**1.85**	2.80	**5.76**
Homomultimeric-link FDP (%)	0.39	0.76	0.32	0.88	0.60	1.18

Supplementary Fig. S2: Acquisition method comparison and IMAC enrichment evaluation.

(A) Comparison of acquisition strategies shows that standard XL acquisition outperforms Real-Time Library Search (RTLS). Data were acquired on an Orbitrap Eclipse mass spectrometer using PhoX-enriched cross-linked peptides from *Arabidopsis* cell lysates and searched against the lysate proteome protein database. RTLS acquisition reduced the identification of monolinks, defined as species in which one NHS ester reacts with a peptide while the other is hydrolyzed, by approximately 48%, and loop-links, defined as cross-links formed within a single peptide, by about 31%. However, RTLS also markedly decreased the detection of inter peptide cross-links, including both intra-protein (intralinks) and inter-protein (interlinks) interactions, by 42% and 41%, respectively.

(B) Percentage analysis of detected peptides indicates efficient enrichment using the IMAC workflow, with regular non phospho-modified linear peptides comprising less than 12% of all identifications. Notably, because precursor ions with charge states of 3+ or higher were preferentially selected for MS/MS analysis, the true proportions of both regular peptides and phosphopeptides may be slightly underestimated. For simplicity, only the peptide backbone sequence, excluding PTM modifications and cross-linking sites, was considered, and unique peptide counts are reported. The relative abundance trends, from most to least abundant, are consistent across cell lysate, chloroplast, and nucleus samples. All data were derived from pLink 3.2 searches, except for phosphopeptide identifications, which were obtained using FragPipe.
(C) False discovery proportion (FDP) analysis at the peptide-pair and PPI levels was performed using a human entrapment library to estimate the FDP. Separate entrapment databases were generated for each dataset and searched independently using pLink 3.2.

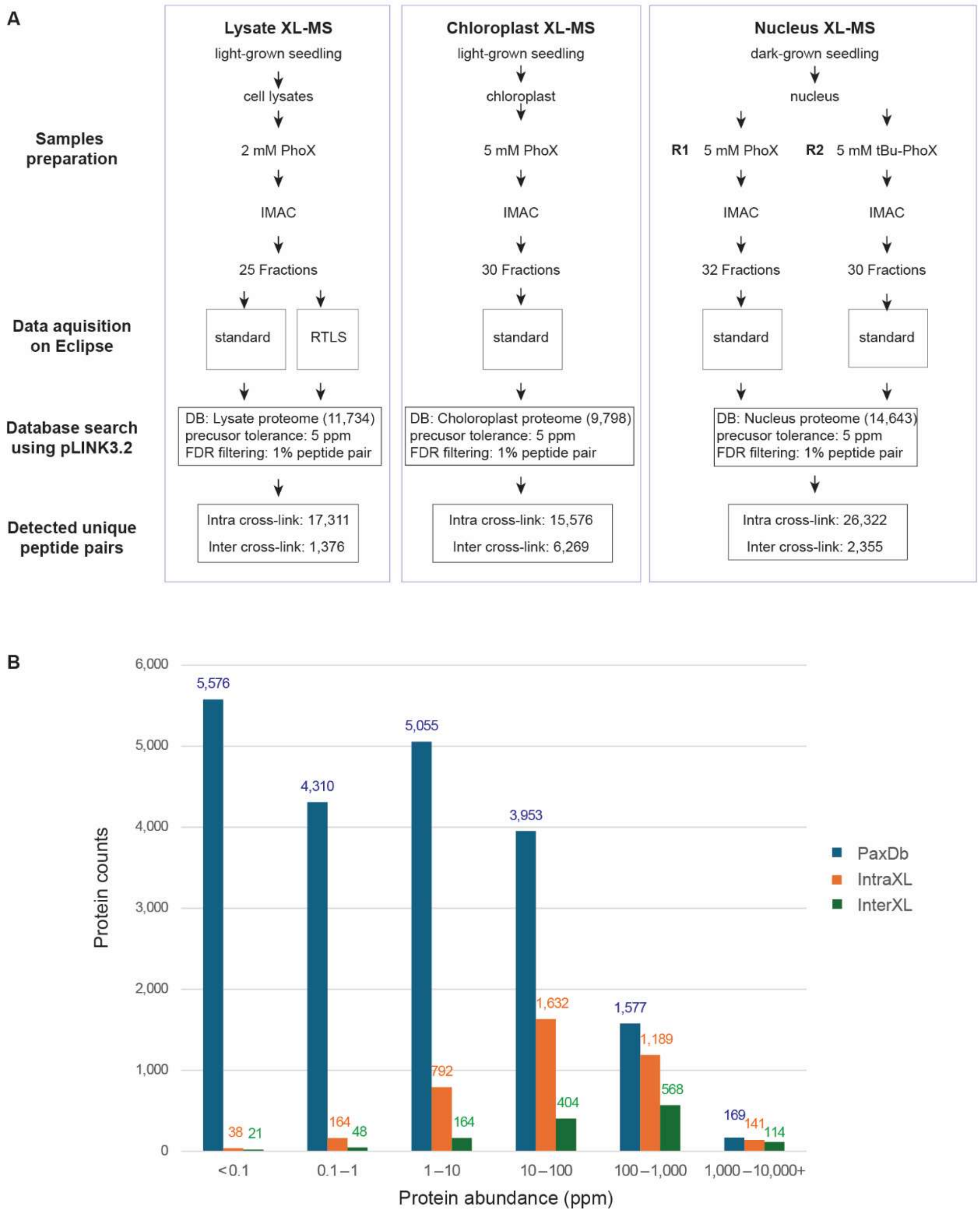

Supplementary Fig. S3: Summary of XL-MS experiments.
(**A**) Key details of sample preparation, data acquisition using the Orbitrap Eclipse mass spectrometer, and database searching using pLink3.2 are provided. A summary of the unique cross-linked peptide pairs identified across experiments is also included. The peptide-pair is defined by distinct peptide backbone sequence, and cross-linking sites, regardless of charge states and variable modifications.

(**B**) The distribution of cross-linked protein identifications across abundance ranks, based on the PaxDb index (pdx-dp.org; blue), indicates broad proteome coverage. In addition to highly abundant proteins, a substantial number of medium- and low-abundance proteins were also identified as cross-linked for both intra-protein (orange) and inter-protein (green) crosslinks, highlighting the depth of the approach. The x-axis represents protein abundance, expressed in ppm (parts per million), reflecting the relative abundance of each protein compared to all others in the sample, while the y-axis shows the counts.

A

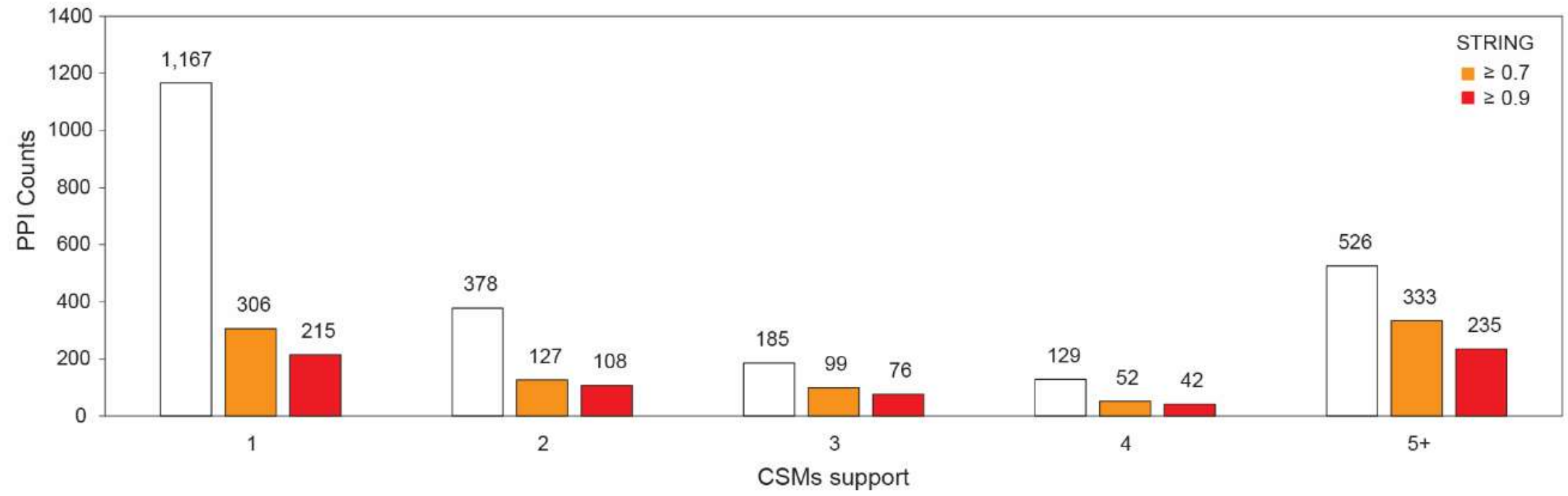

B

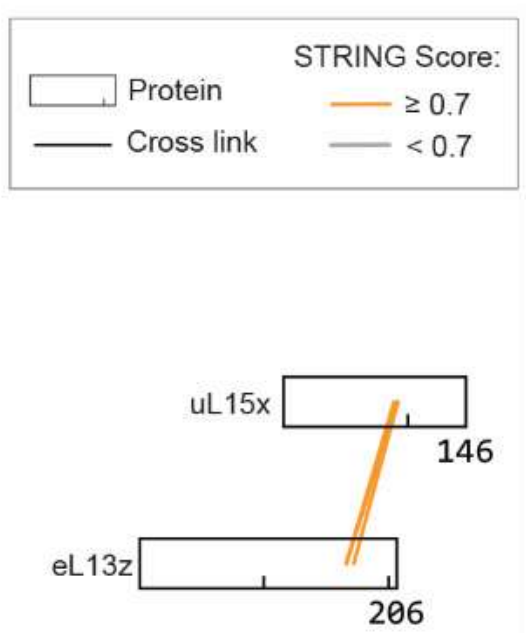

Protein1	Linkage site	PepSeq1	Protein2	Linkage site	PepSeq2	Peptide_Mass
uL15x	K89	AKSTKDNVPLIDVTQHGFFK	eL13z	K166	EKPTMELVKLTSEMK	4218.090052
uL15x	K92	AKSTKDNVPLIDVTQHGFFK	eL13z	K166	EKPTMELVKLTSEMK	4218.090052
uL15x	K92	STKDNVPLIDVTQHGFFK	eL13z	K166	EKPTMELVKLTSEMK	4018.957986
uL15x	K92	AKSTKDNVPLIDVTQHGFFK	eL13z	K172	LTSEMKSFK	3524.716976
uL15x	K92	STKDNVPLIDVTQHGFFK	eL13z	K172	LTSEMKSFK	3325.58491
uL15x	K92	STKDNVPLIDVTQHGFFK	eL13z	K172	LTSEMKSFKAFDK	3786.812323
uL15x	K92	STKDNVPLIDVTQHGFFK	eL13z	K172	LTSEMKSFKAFDKIR	4055.997483
uL15x	K92	STKDNVPLIDVTQHGFFKVLGK	eL13z	K172	LTSEMKSFKAFDK	3722.853793

C

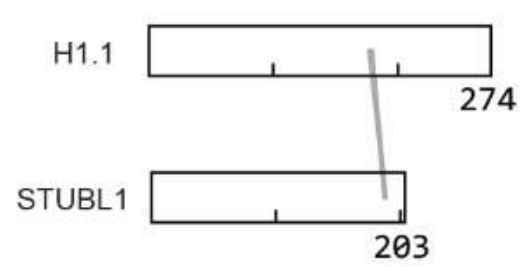

Protein1	Linkage site	PepSeq1	Protein2	Linkage site	PepSeq2	Peptide_Mass
H1.1	K177	TIAVKPK	STBL1	K188	KKVTAK	1639.918193
H1.1	K179	TIAVKPKTAAAK	STBL1	K187	KKVTAK	2082.172153
H1.1	K179	TIAVKPKTAAAK	STBL1	K188	KKVTAK	2082.172153

D

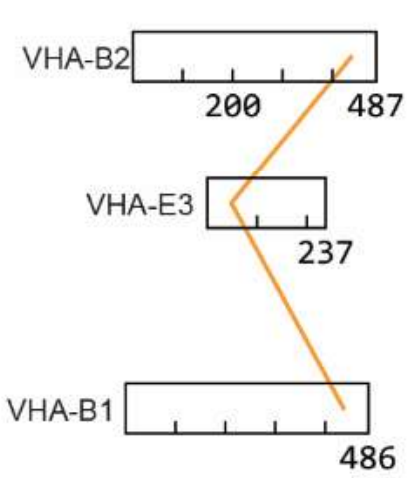

Protein1	Linkage site	PepSeq1	Protein2	Linkage site	PepSeq2	Peptide_Mass
VHA-E3	K48	LQLVEAEKKK	VHA-B1	K437	KFVMQGAYDTR	2710.330892
VHA-E3	K49	LQLVEAEKKK	VHA-B1	K437	KFVMQGAYDTR	2710.330892
VHA-E3	K48	LQLVEAEKKK	VHA-B2	K438	KFVAQGAYDTR	2650.327524
VHA-E3	K49	LQLVEAEKKK	VHA-B2	K438	KFVAQGAYDTR	2650.327524

E

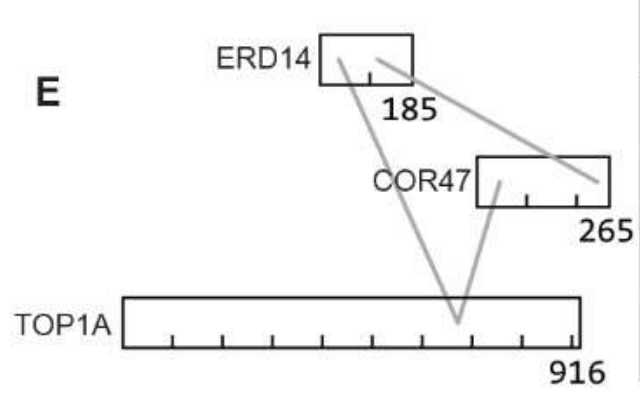

Protein1	Linkage site	PepSeq1	Protein2	Linkage site	PepSeq2	Peptide_Mass
TOP1A	K672	IKFDFLGK	EDR14	K38	KDETKPEETPIASEFEQK	3282.549239
TOP1A	K672	IKFDFLGK	EDR14	K42	KDETKPEETPIASEFEQK	3282.549239
TOP1A	K672	IKFDFLGK	COR47	K46	EEEVKPQETTTLESEFDHK	3452.578539
ERD14	K121	KGFMEKLK	COR47	K244	EKLPGYHAK	2232.09219

F

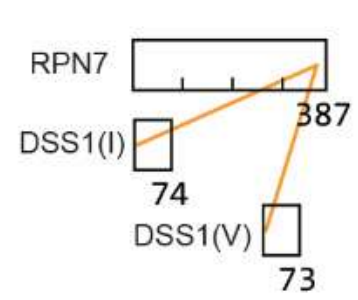

Protein1	Linkage site	PepSeq1	Protein2	Linkage site	PepSeq2	Peptide_Mass
RPN7	K369	NALYQATIKQGDFLLNR	DSS1(I)	K6	AAEPKAATAEVVK	3500.737147
RPN7	K369	NALYQATIKQGDFLLNR	DSS1(V)	K6	AAEPKAAVEVVK	3427.727995

Supplementary Fig. S4. CSM support for PPI identifications and alignment of observed

cross links with site specific and homologous protein regions.

Evidence of identification consistency is shown by redundant mapping to identical sites or conserved regions. Precursor mass, peptide sequence, and cross-linked sites are shown.

(**A**) Distribution of CSM support for PPI identifications. A total of 1,167 heteromultimeric interactions (49%) were supported by a single cross-linked spectral match (CSM), while 51% were supported by multiple CSMs. Only uniquely identified CSMs were included in the analysis, with redundant matches removed. A CSM was defined as distinct based on differences in peptide backbone sequence, cross-linking site, charge state, or variable modifications (e.g., oxidation). STRING scores are provided for comparison. The x-axis represents unique CSM counts, and the y-axis represents PPI counts.

(**B**) Ribosomal subunit cross-links between uL15x K92 and eL13z-K166 or K172, showing variable miscleavages.

(**C**) Interaction between Histone 1.1 K177 or K179 and SUMO targeted ubiquitin E3 ligase 1, STUBL1, at K187 or 188.

(**D**) Vacuolar proton ATPase subunit VHA-E3-K49 linked to homologous sites VHA-B1 K437 and VHA-B2 K438.

(**E**) DNA topoisomerase I alpha, TOP1A, K672 linked to dehydrin homologs ERD14 at K38 or K42 and COR47 at K46.

(**F**) 26S proteasome subunit RPN7-K369 linked to the same sites in homologs DSS1(I) K6 and DSS1(V) K6.

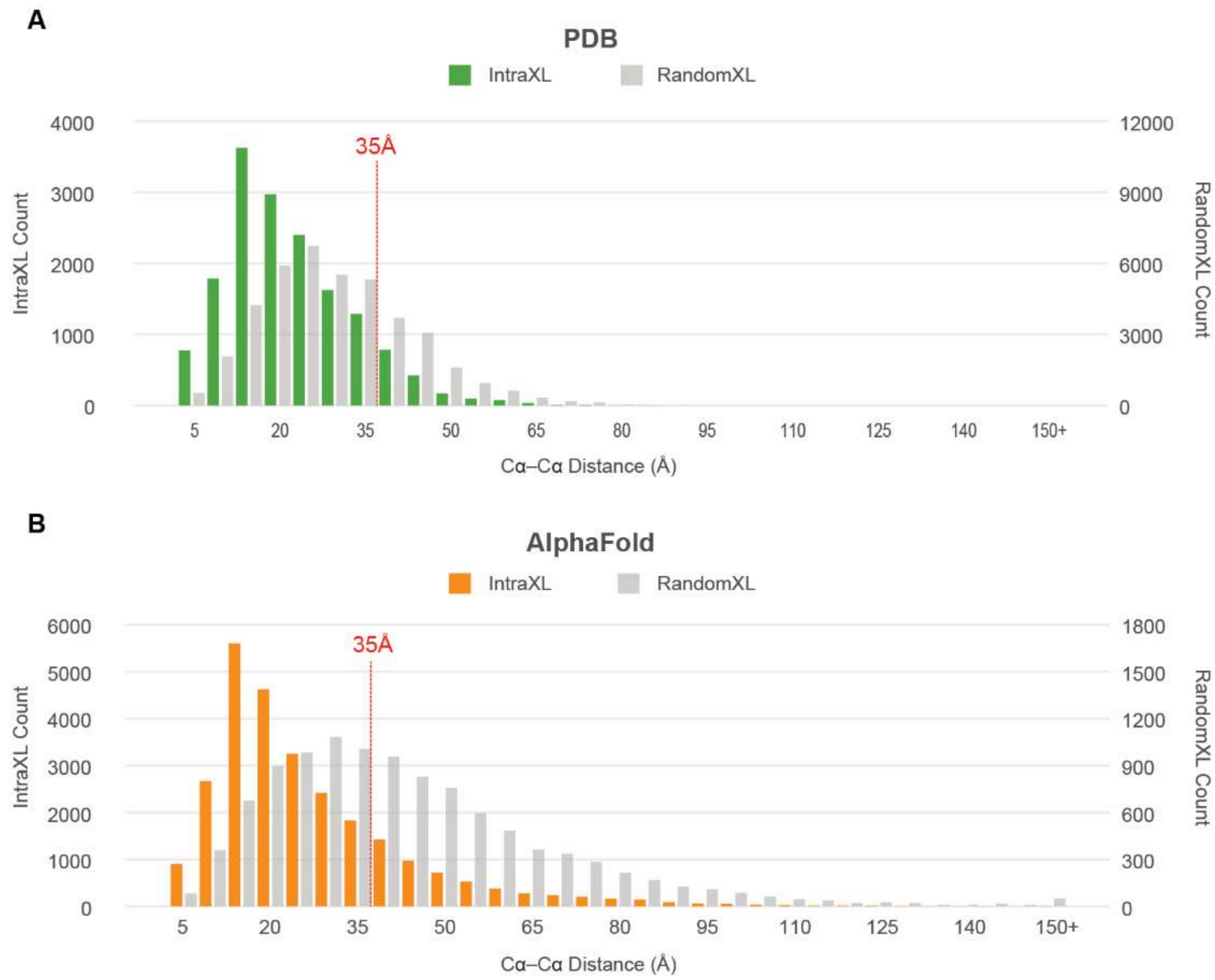

Supplementary Fig. S5. Validation of cross-link distance constraints using PDB and AlphaFold structures.

(**A**–**B**) Distributions of minimum Euclidean distances (Å, Cα–Cα) for identified cross-linked residue pairs compared with randomly generated residue pairs mapped onto Protein Data Bank (PDB) structures (A) and AlphaFold models (B) from *Arabidopsis*. Notably, the mean and maximum distances observed in AlphaFold structures are greater than those in PDB structures, likely reflecting the fact that PDB entries often represent partial protein fragments rather than full-length proteins.

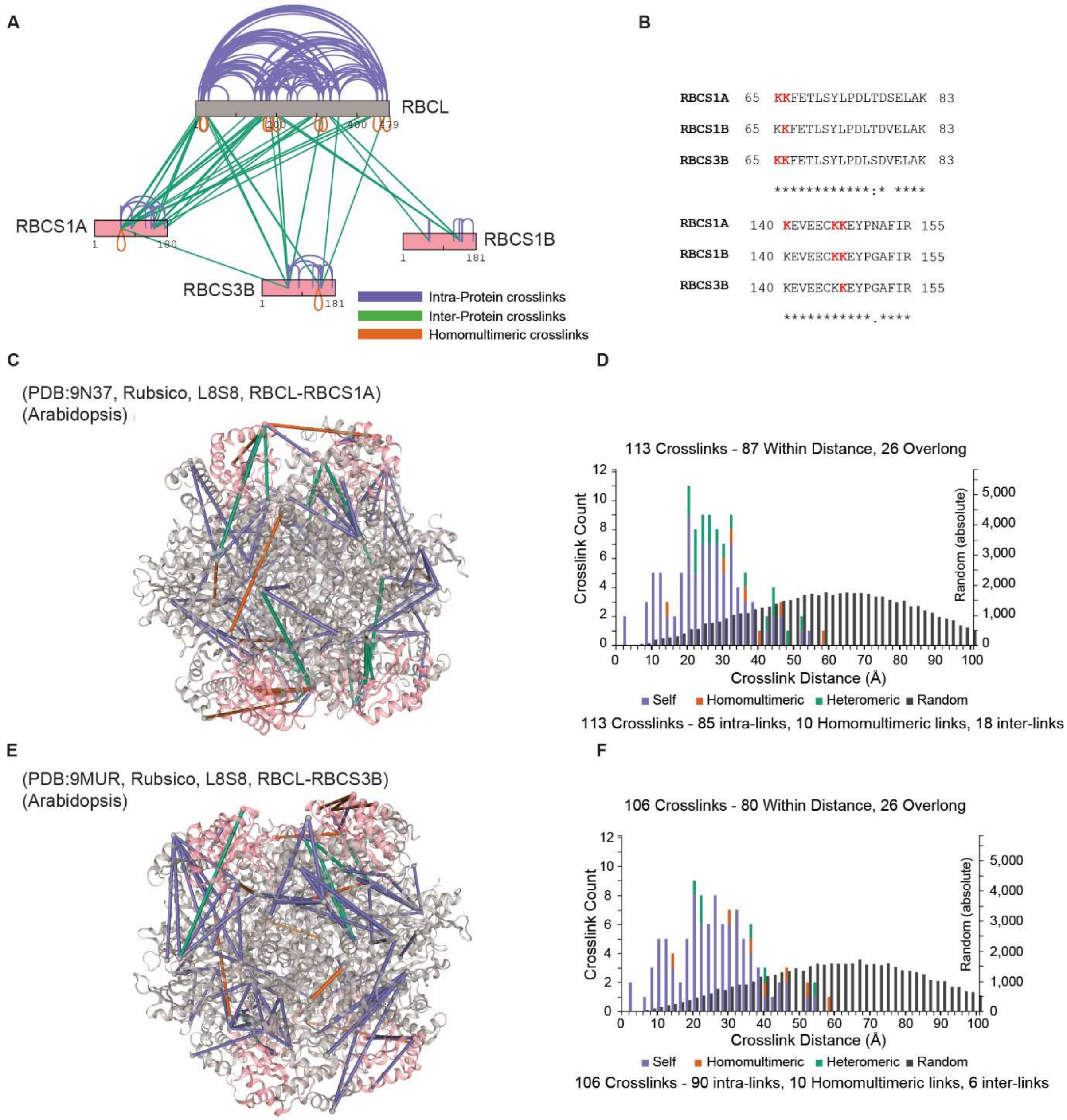

Supplementary Fig. S6. Mapping of Rubisco cross-links on PDB structures of Arabidopsis Rubisco.

Cross-links are color-coded by type: purple for self (intra)links, green for heteromeric (inter)links, and orange for homomultimeric links.

(**A**) Schematic of Rubisco Cross-linking: Diagram illustrating intra-protein cross-links within and inter-protein cross-links between the large subunit (RBCL, grey) and three small subunit isoforms (RBCS1A, 1B, and 3B, pink). RBCS1A is the dominant isoform and therefore yielded the highest number of identified cross-links with RBCL.

(**B**) Sequence Alignment and Isoform-Specific Cross-links: Multiple sequence alignment of RBCS1A, 1B, and 3B highlighting regions (residues 65–83 and 140–155) containing identified

cross-linked peptides with isoform-specific sequence variations. Lysine residues (K), representing cross-linking sites, are highlighted in red. Only peptides with sequence differences are displayed to demonstrate the specificity of isoform assignments. Conservation is indicated by asterisks (*, identical), colons (:, strongly similar), and periods (., weakly similar).
(**C**–**D**) RBCL–RBCS1A Mapping: Cross-links between RBCL and RBCS1A mapped onto the Arabidopsis L_8S_8 Rubisco structure (PDB: 9N37), with the accompanying histogram showing the Cα-Cα distance distribution. RBCL in the 9N37 structure lacks C-terminal region (residues 459-479), reducing cross-link mapping coverage.
(**E**–**F**) RBCL–RBCS3B Mapping: Cross-links between RBCL and RBCS3B mapped onto the Arabidopsis L_8S_8 Rubisco structure (PDB: 9MUR), with the accompanying histogram showing the Cα-Cα distance distribution. RBCL in the 9MUR structure lacks C-terminal region (residue 465-479), reducing cross-link mapping coverage.
.

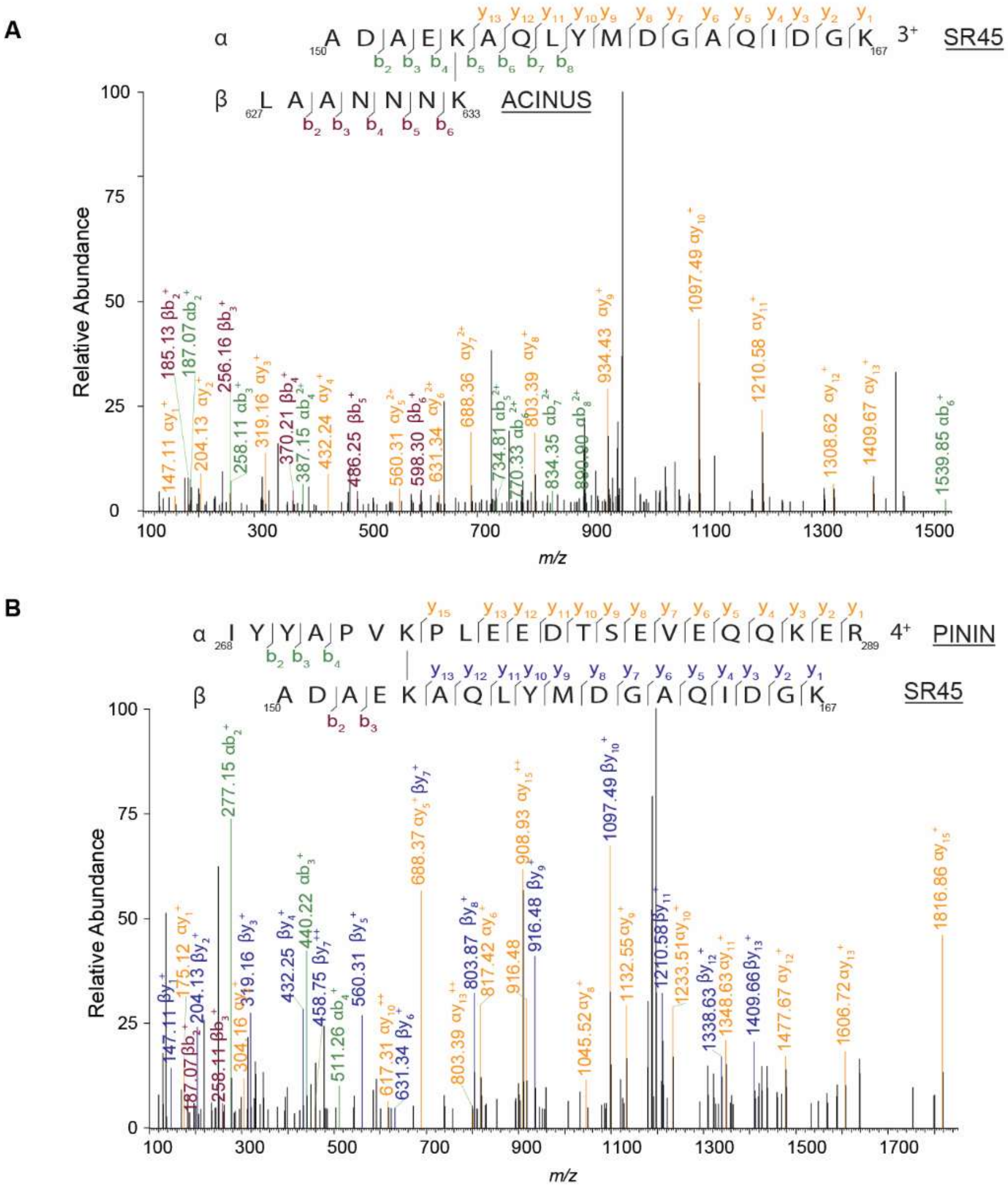

Supplementary Fig. S7. High-confidence identifications of cross-linked peptides between ACINUS–SR45 and PININ–SR45.

b-ions are shown in green and y-ions in yellow. A series of fragment ions, including those from α- and β-peptides, as well as cross-linker-containing ions, were detected, supporting the reliability of the cross-link identifications.

(**A**) Spectrum of the cross-linked peptide between ACINUS-K633 and SR45-K154.

(**B**) Spectrum of the cross-linked peptide between PININ-K274 and SR45-K154.

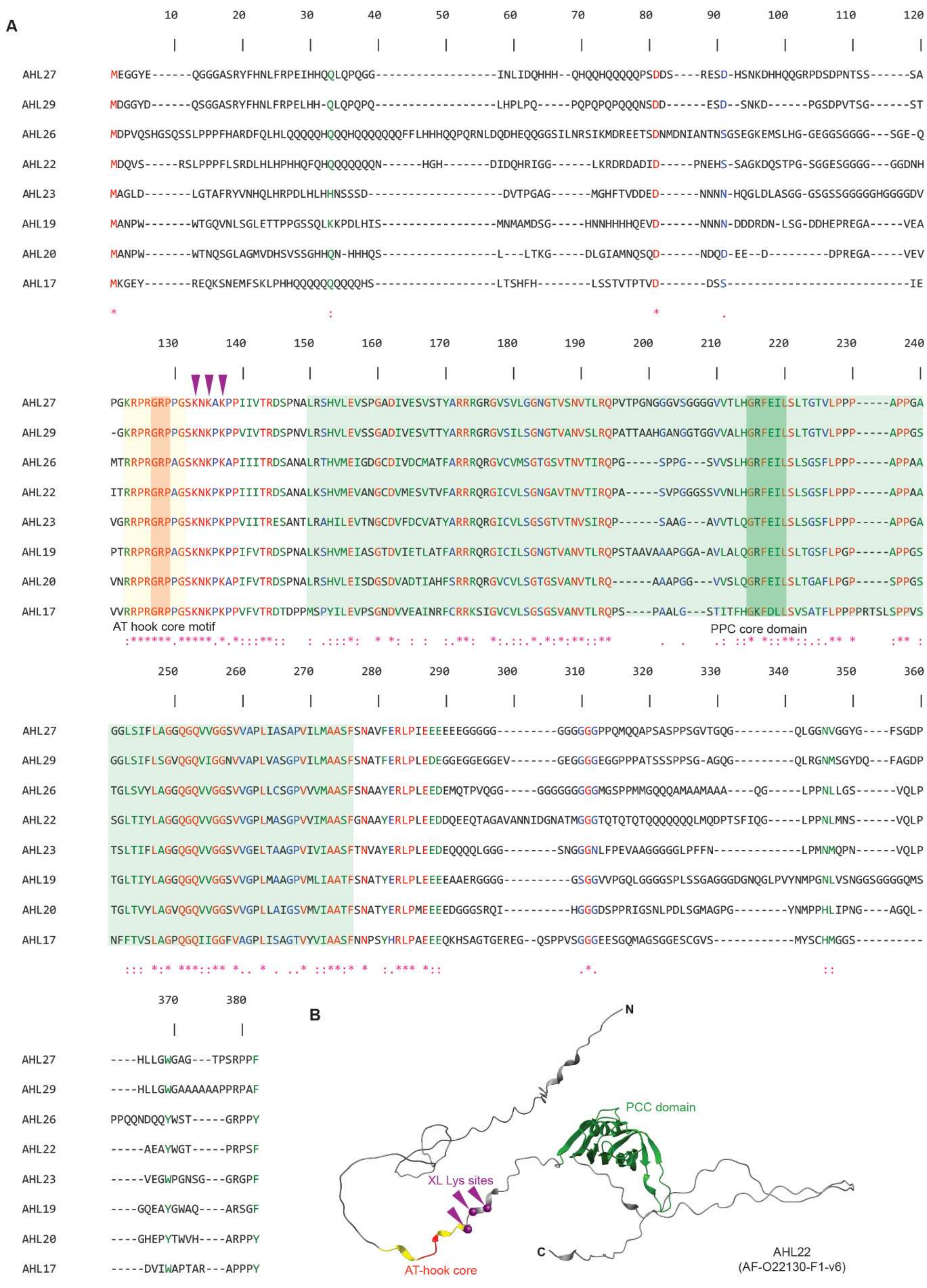
A
B
AHL27
AHL29
AHL26
AHL22
AHL23
AHL19
AHL20
AHL17
AT hook core motif
PPC core domain
N
PCC domain
XL Lys sites
AT-hook core
C
AHL22
(AF-O22130-F1-v6)

Supplementary Fig. S8. Sequence Alignment of the AHL Family and AlphaFold Structural Modeling of Crosslinked Lysine Sites.

(**A**) Multiple Sequence Alignment of AHL Family Members. The alignment was performed using ClustalW. The AT-hook core motif and the Plant and Prokaryote Conserved (PPC) domain (including the core domain) are highlighted in yellow and green, respectively. Within these regions, the core GRP is indicated in purple, and the core GRFEIL is shown in dark green. Red arrows denote the positions of crosslinked lysine residues.

(**B**) AlphaFold Structural Prediction of AHL22. The predicted structure highlights the AT-hook core in yellow and the PPC structured domain in green. Three specific crosslinked lysine sites are indicated by arrows.

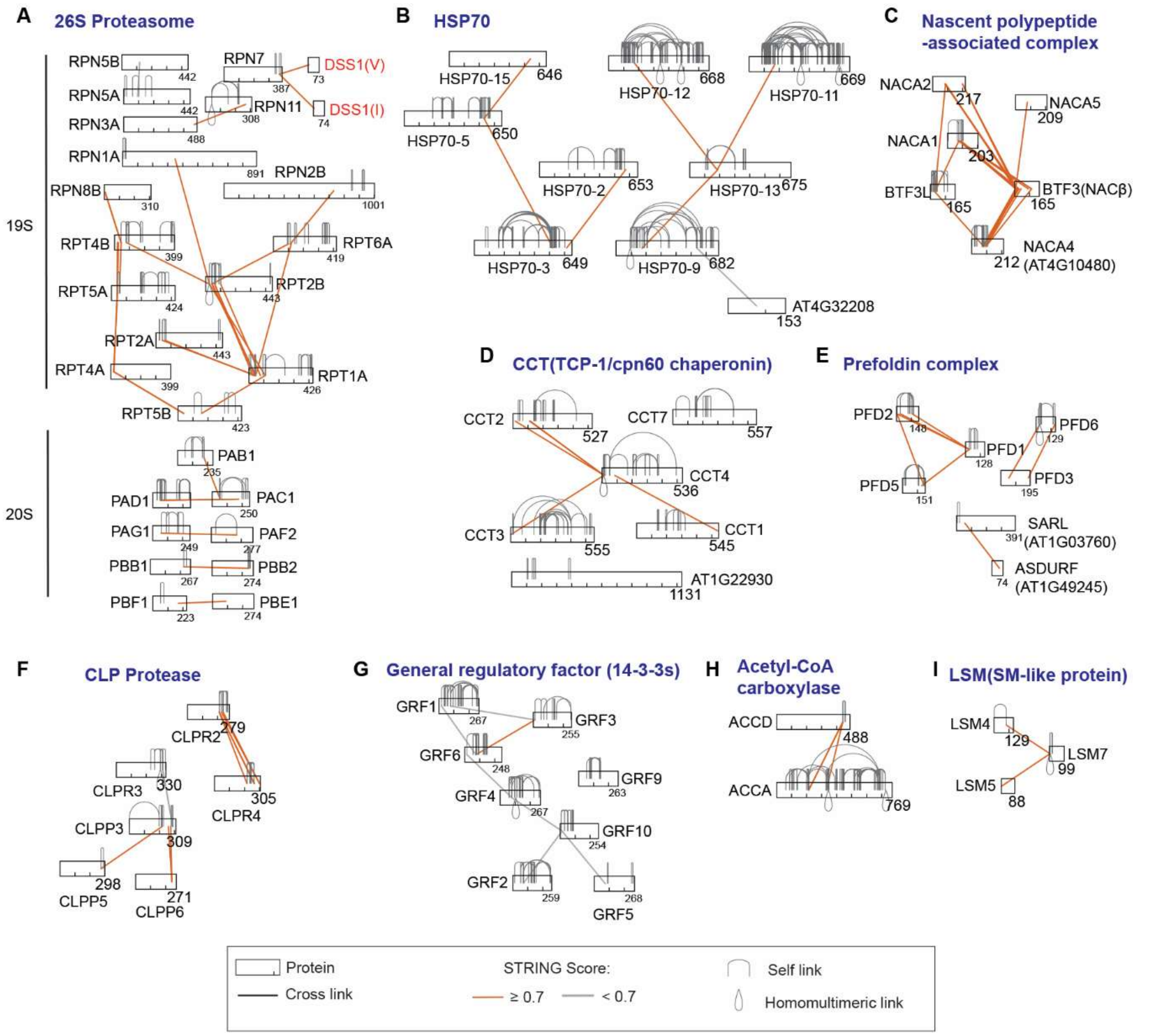

Supplementary Fig. S9. Topological mapping of multiple protein complexes by XL-MS. Proteins are represented as rectangles scaled according to their respective lengths. Cross-links are color-coded based on STRING confidence score thresholds, highlighting both known and novel interactions.

(**A**) Topological mapping of the 26S proteasome complex, comprising the 19S and 20S subunits, showing connectivity among subunits. Interaction interfaces between RPN7 and DSS1(I) and DSS1(V) are highlighted. DSS1 proteins are particularly challenging to resolve by cryo-EM because of their small size, intrinsic disorder, and dynamic interaction properties.

(**B–I**) Topological mapping of multiple protein complexes, including several chaperone-related complexes such as Heat Shock Protein 70 (HSP70), the Nascent Polypeptide-Associated Complex (NAC), CCT (TCP-1/cpn60 chaperonin), Prefoldin (PFD), CLP protease, and General Regulatory Factors (14-3-3 proteins). Additional complexes include Acetyl-CoA Carboxylase (ACC) and the Sm-like small nuclear ribonucleoprotein (LSM) complexes.

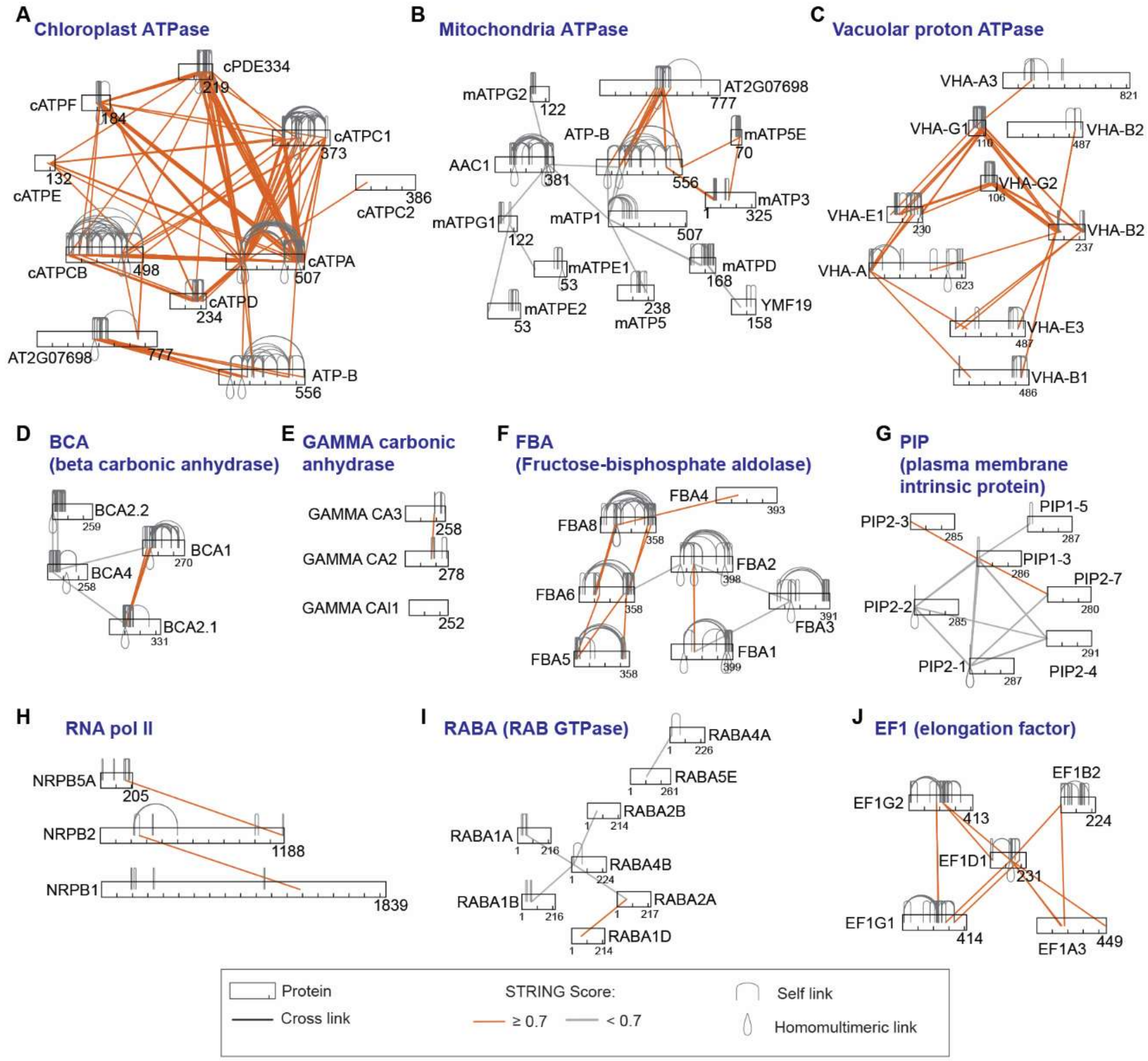

Supplementary Fig. S10. Topological mapping of multiple protein complexes by XL-MS.
(**A**-**C**) Topological mapping of ATPase complexes from chloroplasts, mitochondria, and vacuolar proton ATPase. AT2G07698, encoding the ATPase F1 complex α-subunit, and ATP-B (AT5G08670), both nuclear-encoded proteins, formed cross-links with chloroplast-encoded ATPase complex subunits as well as mitochondria-encoded ATPase subunits, suggesting dual localization and assembly in both organelles.
(**D**-**E**) Topological connections of β- and γ-carbonic anhydrases.
(**F**-**J**) Topological mapping of multiple protein complexes, including fructose-bisphosphate aldolase, plasma membrane intrinsic protein complexes, RNA polymerase II, RAB GTPase, and elongation factor complexes.

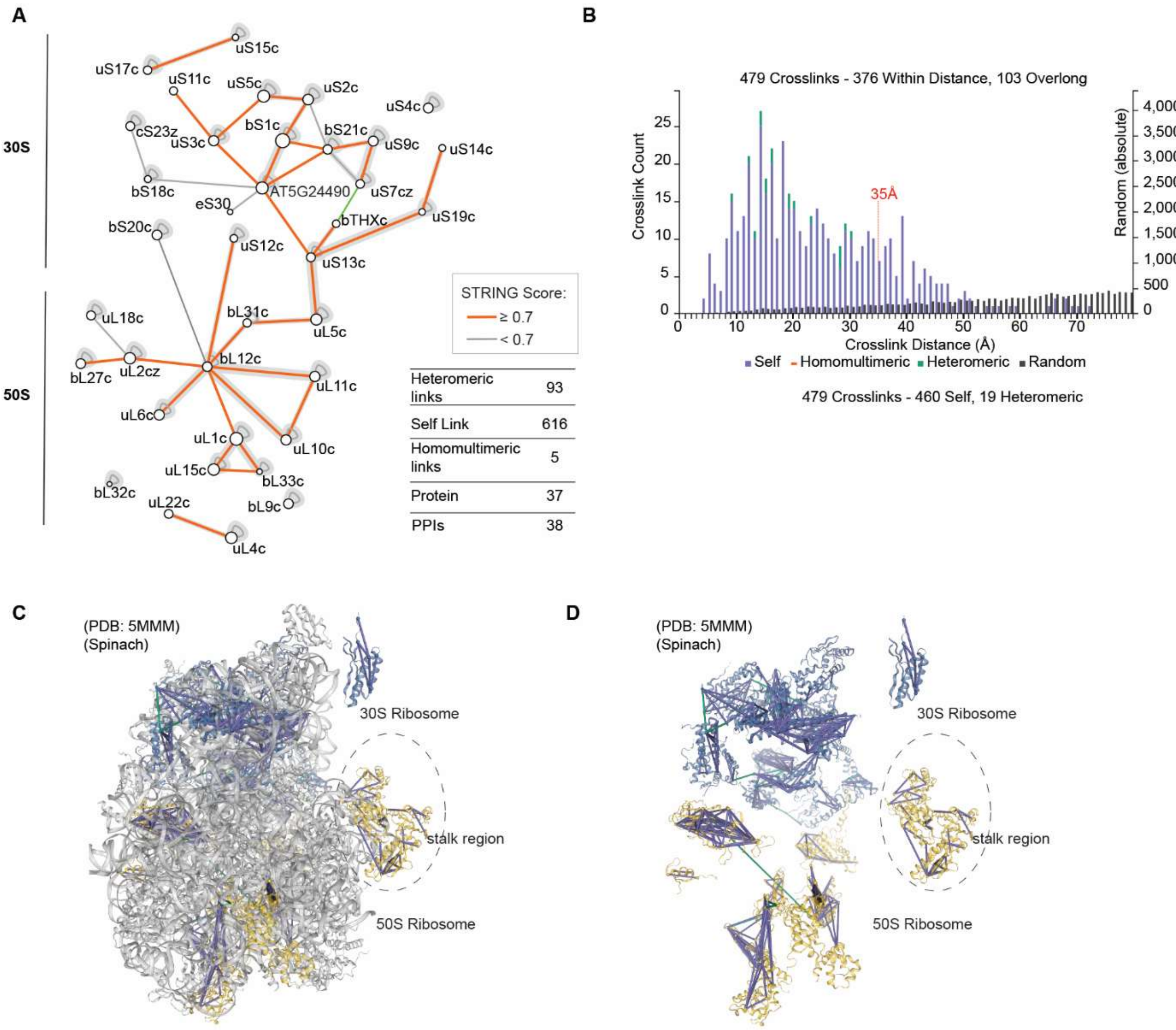

Supplementary Fig. S11. Mapping of cross-links in the chloroplast 70S ribosome complex onto the PDB structure of spinach 70S ribosomes.

(**A**) Topological mapping of cross-links within the 70S ribosome complex.

(**B**–**D**) A total of 479 cross-links were mapped onto the spinach cryo-EM structure (PDB: 5MMM), which lacks bL12c in the stalk region. The structure contains both ribosomal subunits and associated ribosomal RNAs. In (D), ribosomal RNAs were removed to facilitate clearer visualization of the cross-links. The accompanying histogram in (B) shows the distribution of Cα–Cα distances for the mapped cross-links.

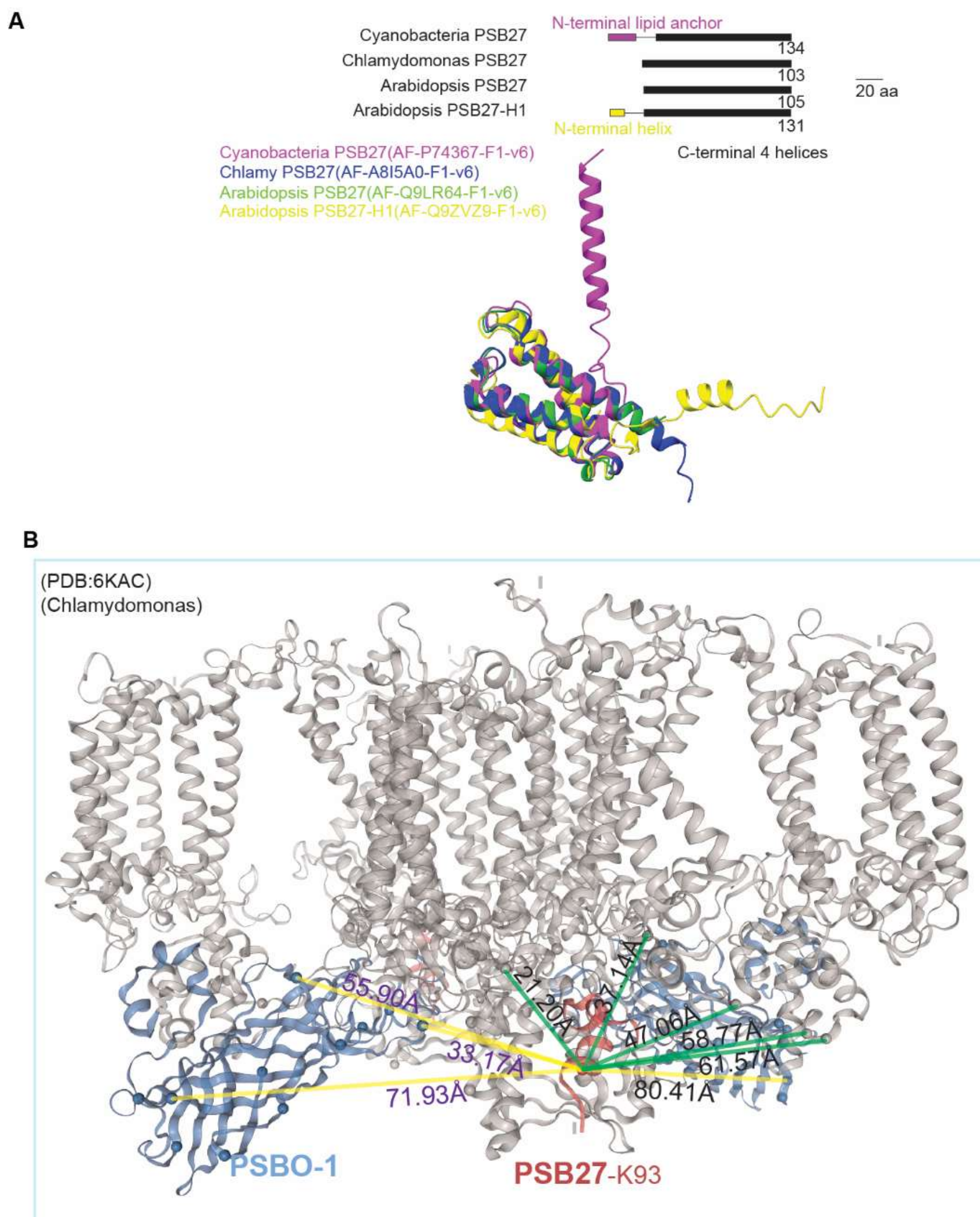

Supplementary Figure S12. Mapping of PSB27 cross-links onto PDB structure 6KAC from Chlamydomonas.

(**A**) Superimposed AlphaFold structures of PSB27 homologs reveal unique structural features of Arabidopsis PSB27. Although Arabidopsis PSB27 retains the conserved C-terminal four-helix bundle, it lacks the lipid anchor and displays divergence in both sequence and surface charge distribution.

(**B**) Cross-links were mapped onto the Chlamydomonas PSII structure (PDB: 6KAC), which contains residues 91–114 of PSB27. PSB27 is shown in purple, PSII core subunits in gray, and the PSBO-1 protein of the oxygen-evolving complex (OEC) subunits in blue. Yellow lines indicate cross-links between PSB27 and PSBO-1, whereas green lines indicate cross-links between PSB27 and PSII core subunits (PsbA, PsbB, PsbC, and PsbD). The observed distance constraints suggest a dynamic interaction between PSB27 and the PSII complex.

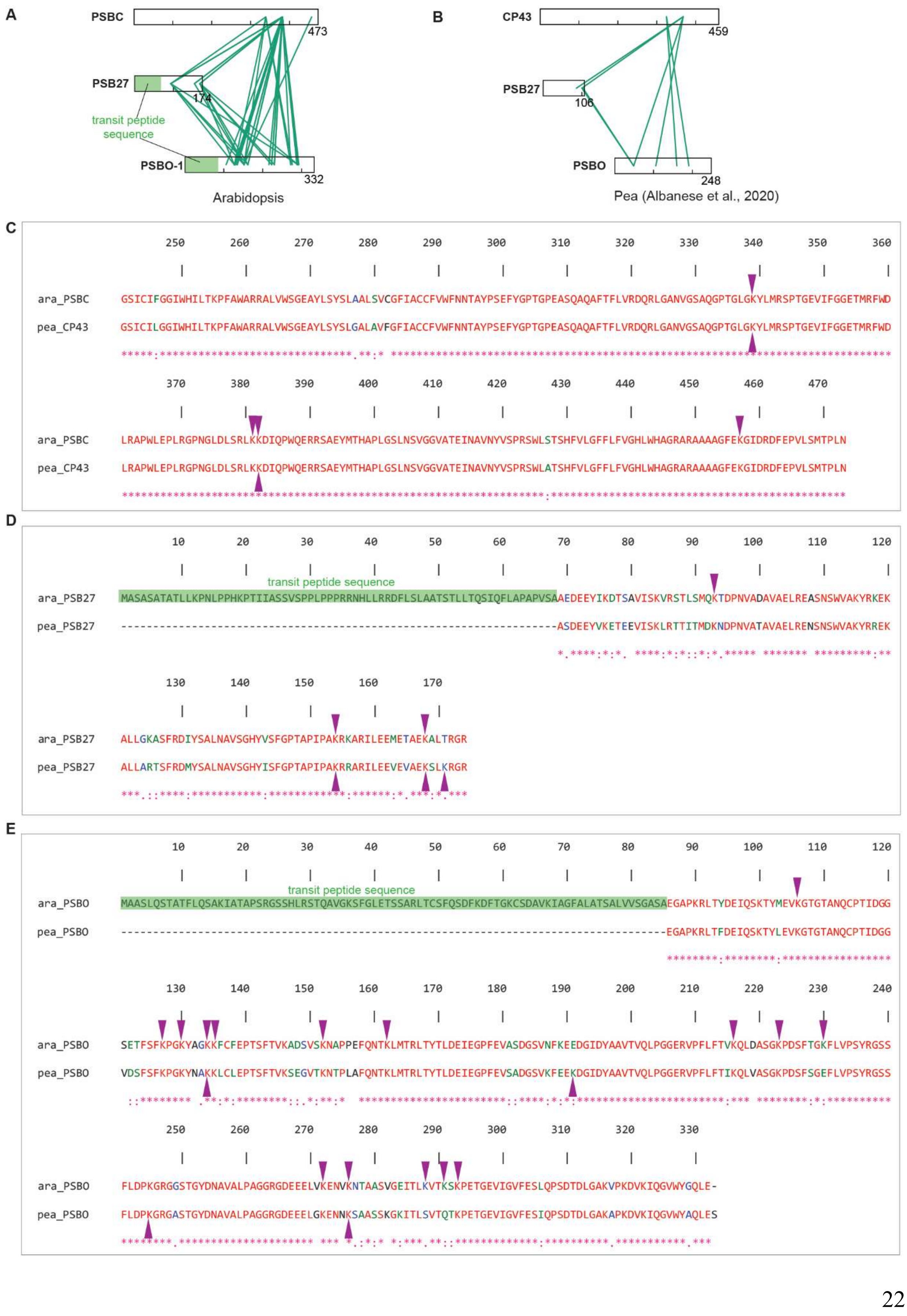

A
PSBC
473
PSB27
174
transit peptide sequence
PSBO-1
332
Arabidopsis
B
CP43
459
PSB27
106
PSBO
248
Pea (Albanese et al., 2020)
C
ara_PSBC
pea_CP43
D
transit peptide sequence
ara_PSB27
pea_PSB27
E
transit peptide sequence
ara_PSBO
pea_PSBO

Supplementary Fig. S13. Comparison of cross-linked sites among PSB27, PSBC, and PSBO between Arabidopsis and pea.

(**A**–**B**) Detected cross-links between PSB27 and PSBO, and between PSB27 and PSBC, in Arabidopsis and pea (Albanese, 2020).

(**C**–**D**) Comparison of the detected cross-linked sites (indicated by arrows) between the two species, revealing conserved interaction sites. Additional cross-linked sites were identified in the present study.

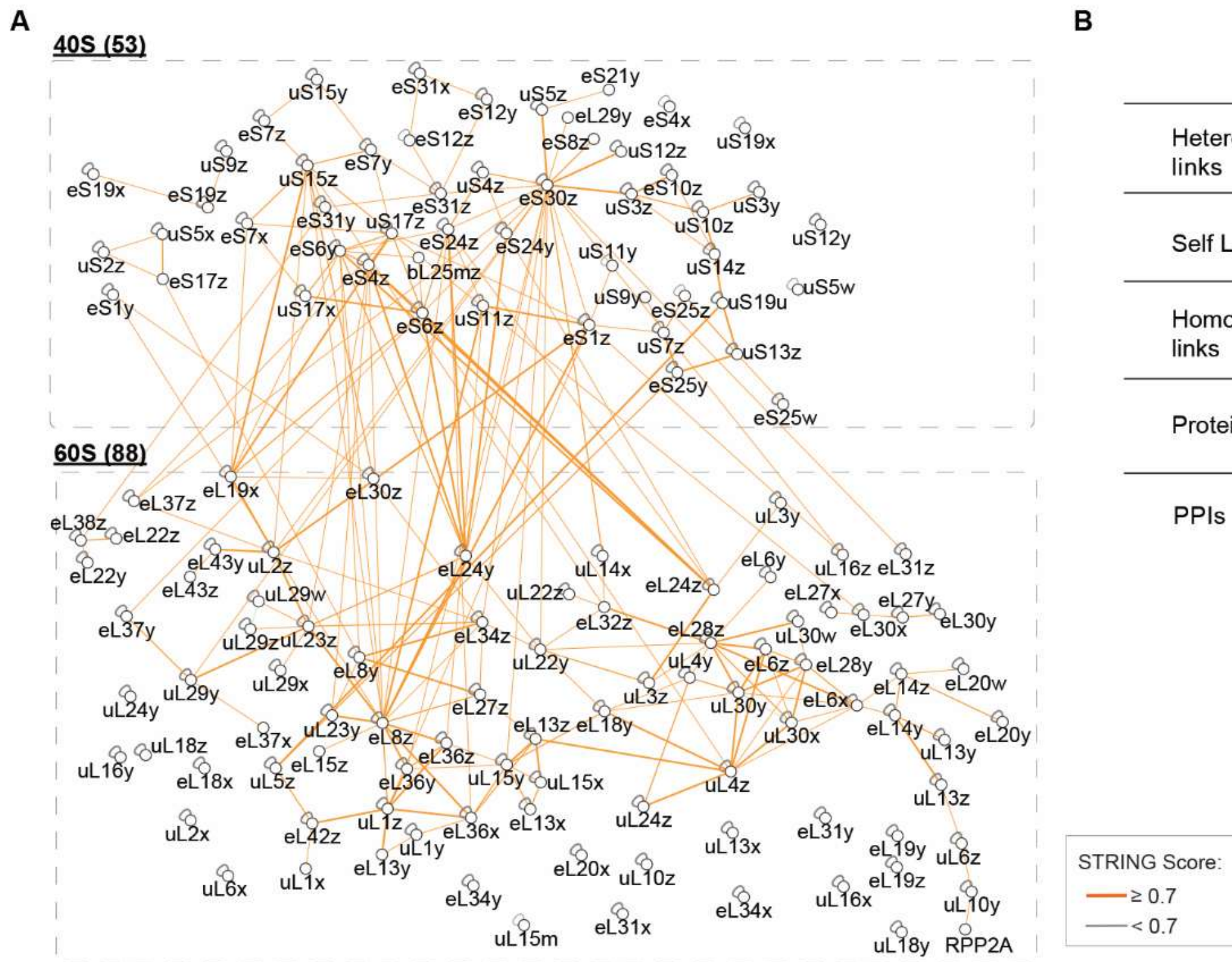

B

Heteromeric links	513
Self Link	4053
Homomultimeric links	128
Protein	141
PPIs	224

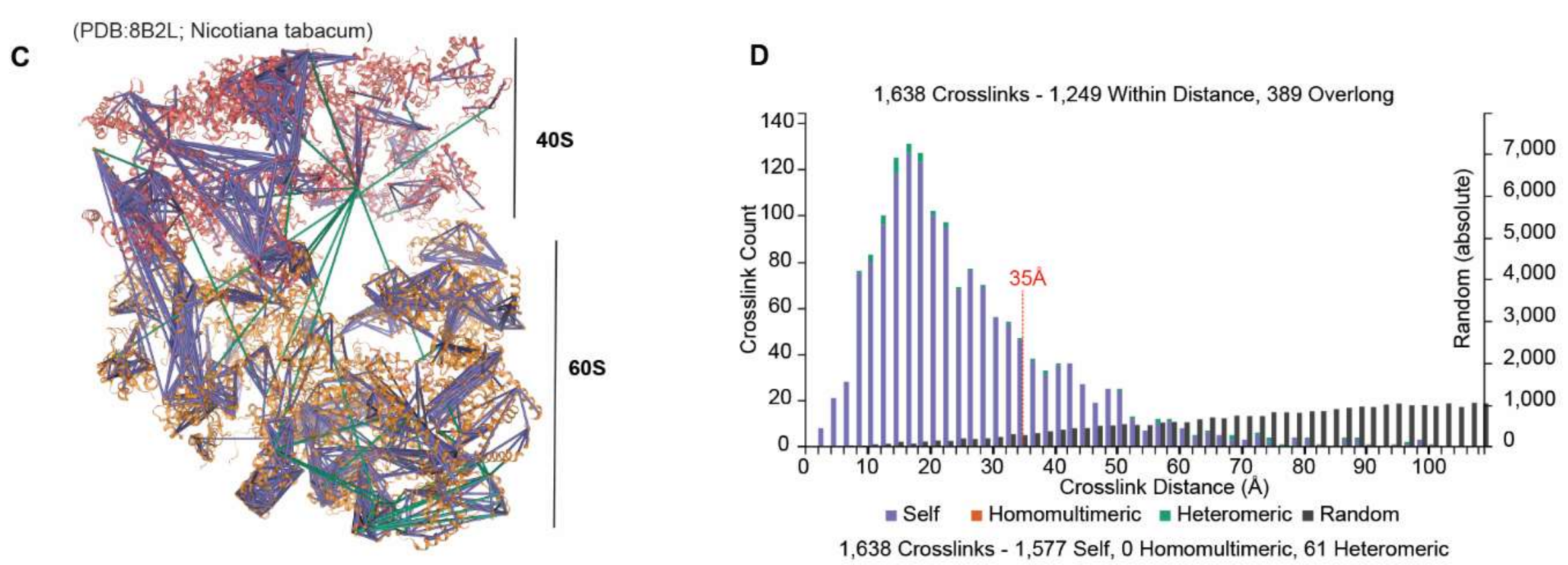

Supplementary Fig. S14. Topology of the 80S ribosomal complex.
(**A**) Topological arrangement of the 40S and 60S ribosomal subunits based on their connectivity. Links between proteins are colored according to the confidence thresholds of the STRING score.
(**B**) Summary of cross-links and protein-protein interaction (PPI) information from (A).
(**C**) 80S ribosome cross-links mapped onto tobacco 80S ribosome (PDB: 8B2L). The large ribosomal subunit is colored orange and the small ribosomal subunit is colored red. Links are color-coded according to link type: purple for self-links (intralinks), green for heteromeric links (interlinks), and orange for homomultimeric links. Ribosomal RNAs were removed to facilitate clearer visualization of the cross-links
(**D**) Distribution of cross-link distances from the integrated cross-link model in (C).

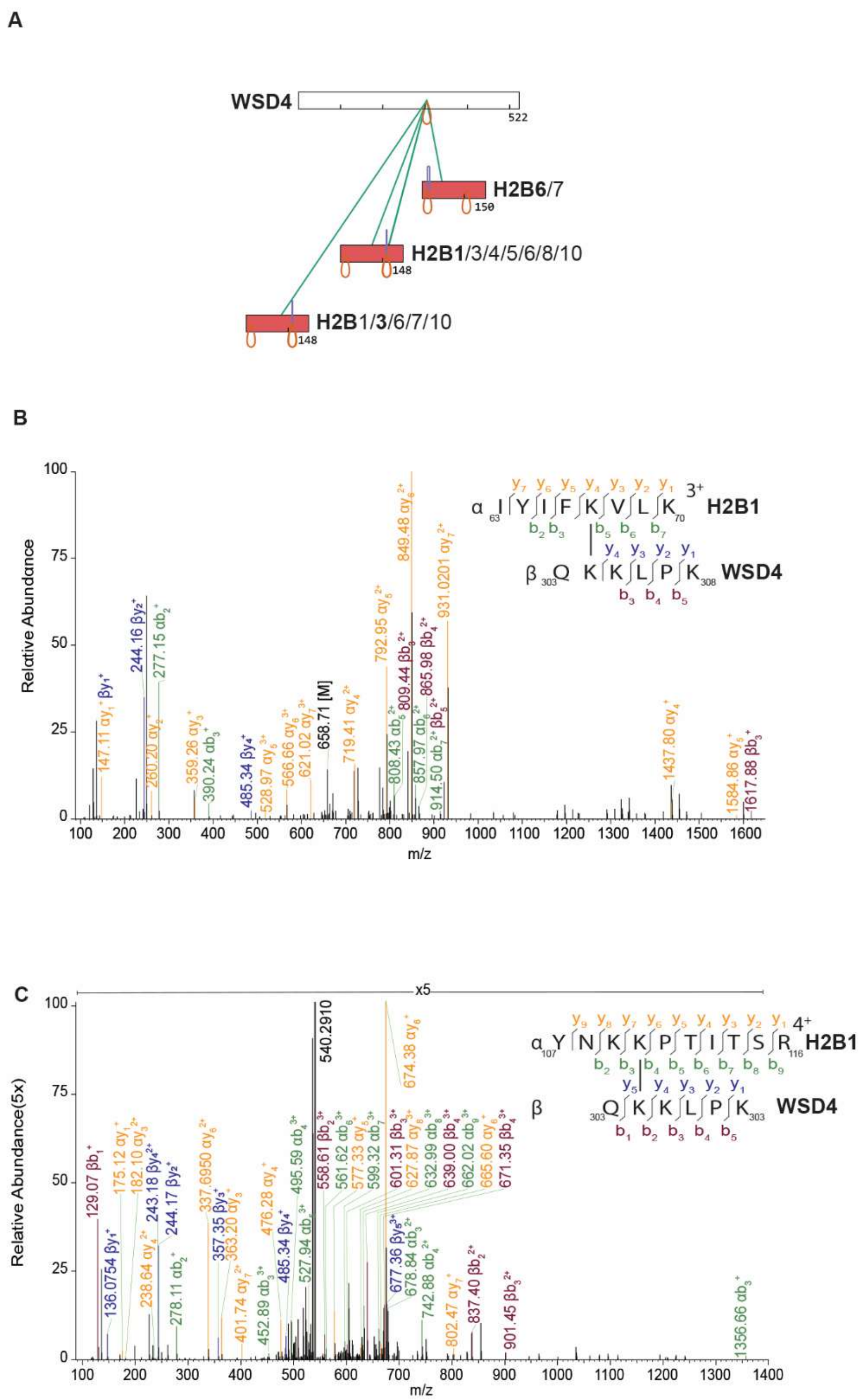
A
WSD4
522
H2B6/7
150
H2B1/3/4/5/6/8/10
148
H2B1/3/6/7/10
148
B
Relative Abundance
m/z
658.71 [M]
α 63 I Y I F K V L K 70 3+ H2B1
β 303 Q K K L P K 308 WSD4
C
x5
Relative Abundance(5x)
m/z
540.2910
α 107 Y N K K P T I T S R 116 4+ H2B1
β 303 Q K K L P K 303 WSD4

D

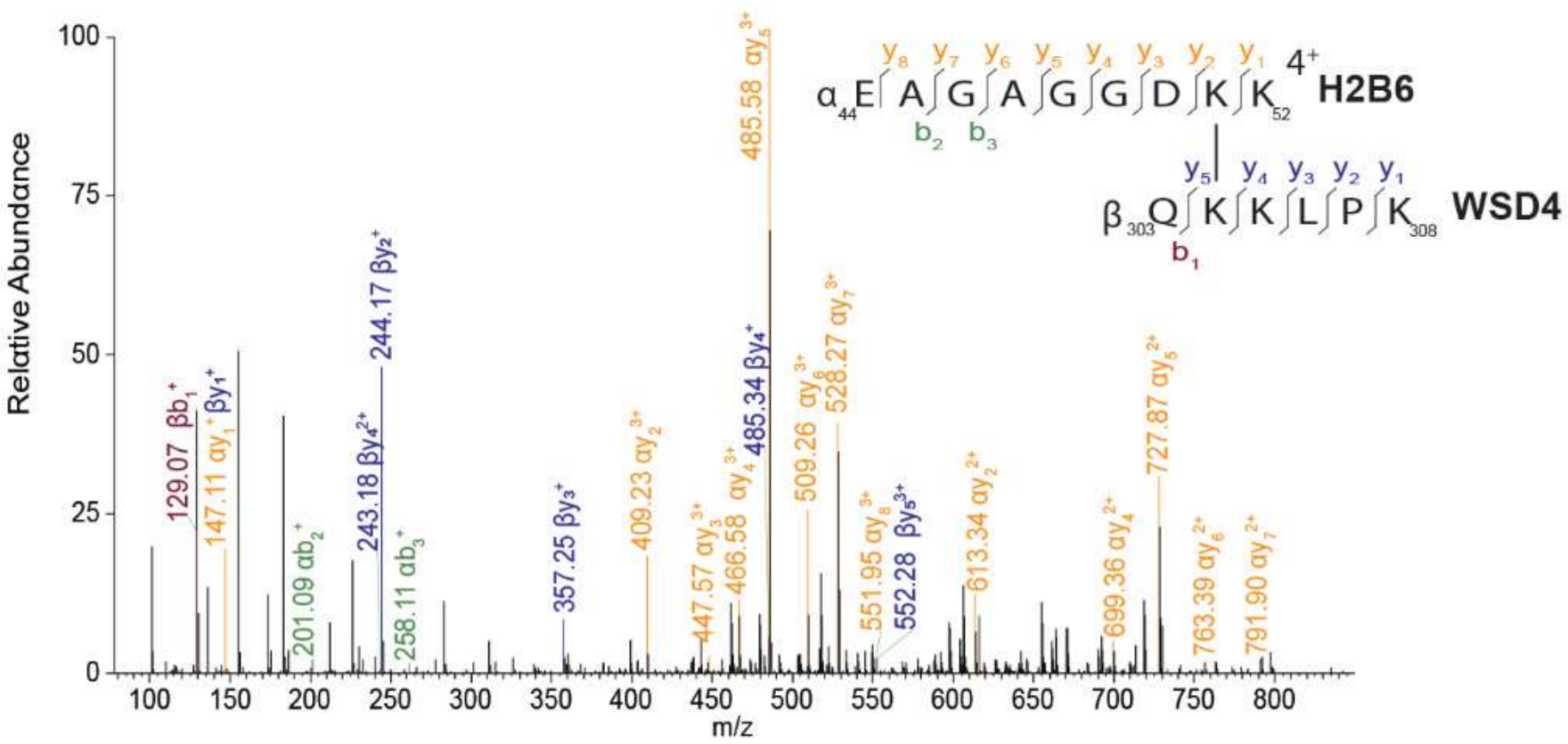

E

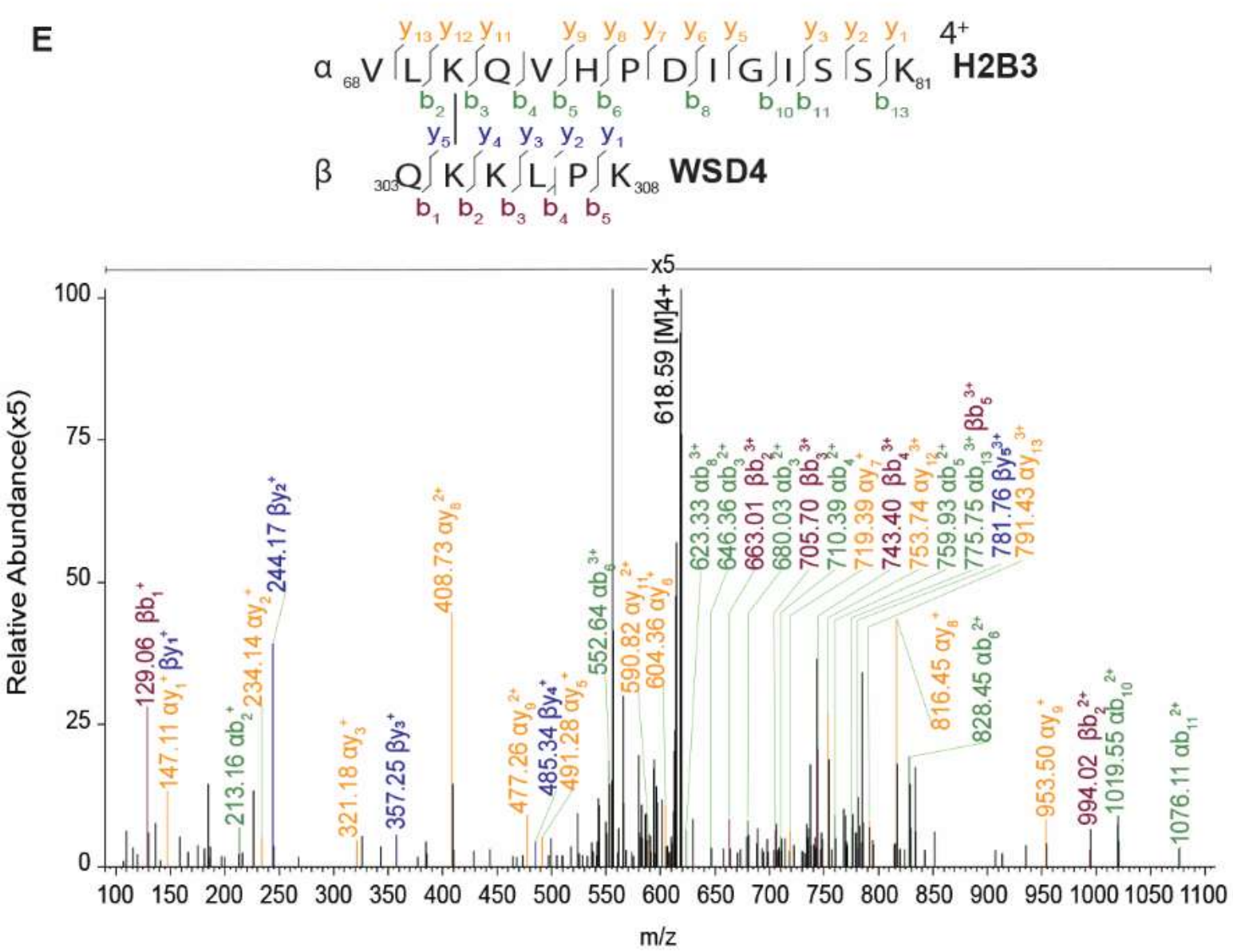

Supplementary Fig. S15. XL-MS identifies WSD4 as a novel histone interactor associated with H2B family members.

(**A**) Cross-links were detected between WSD4, an O-acyltransferase, and H2B family members. Owing to the high sequence homology among H2B family proteins, several identified peptides mapped to shared H2B sequences.

(**B–E**) Representative cross-link spectra identified by pLink3.2 demonstrating interactions between WSD4 and H2B family members. Multiple series of fragment ions, including α- and β-peptide ions as well as cross-linker-containing fragment ions, were detected, supporting the confidence and reliability of the cross-link identifications.

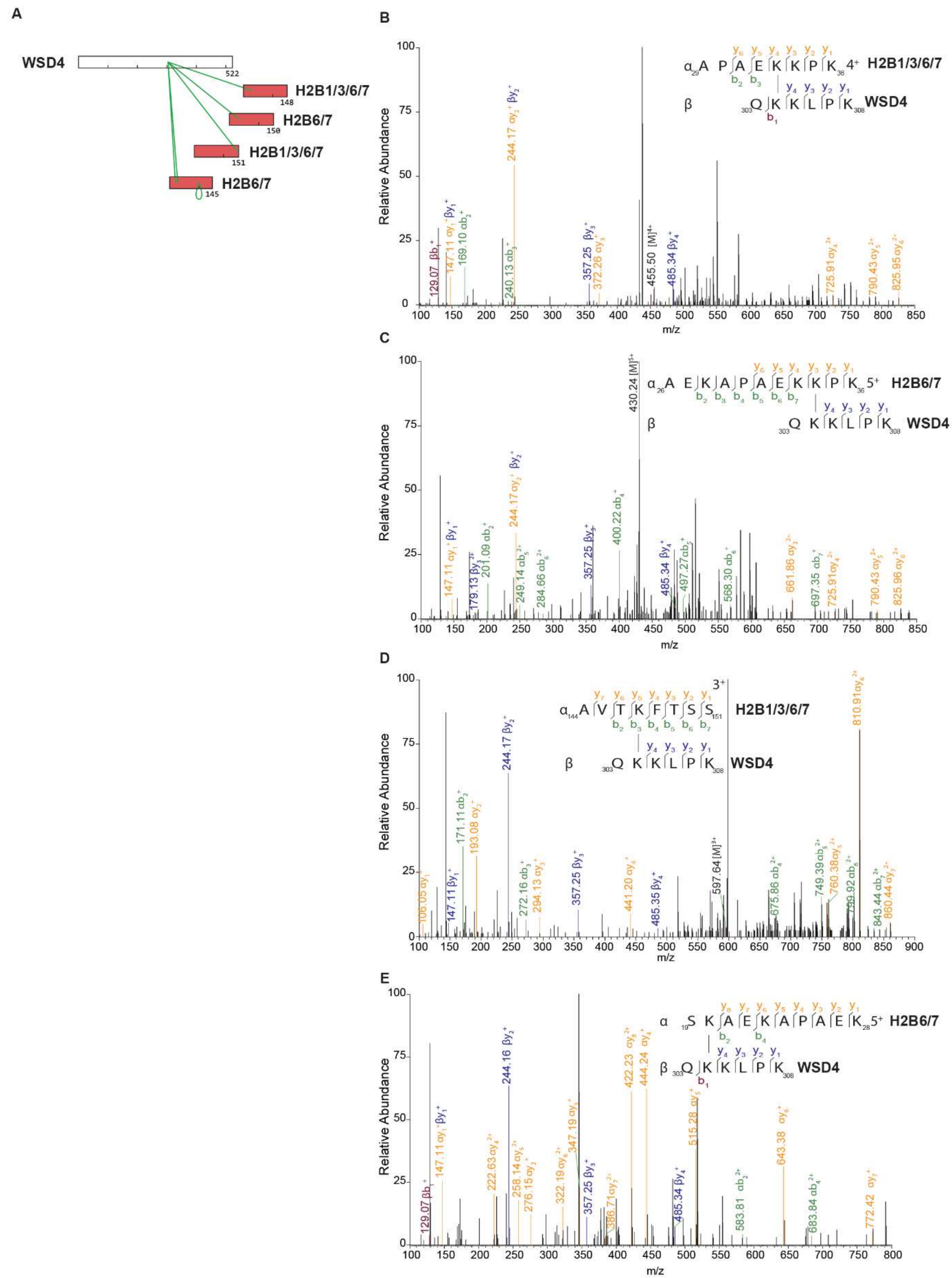

Supplementary Fig. S16. Supplementary Fig. S16. XL-MS identifies WSD4 as a novel histone interactor associated with H2B family members using pLink2.

(**A-E**) Cross-linked peptides identified by pLink2.3.11 at a 5% FDR at the CSM level support the PPI between H2B family members and WSD4. These peptides were not identified by pLink3.2.0 at a 1% peptide-level FDR.

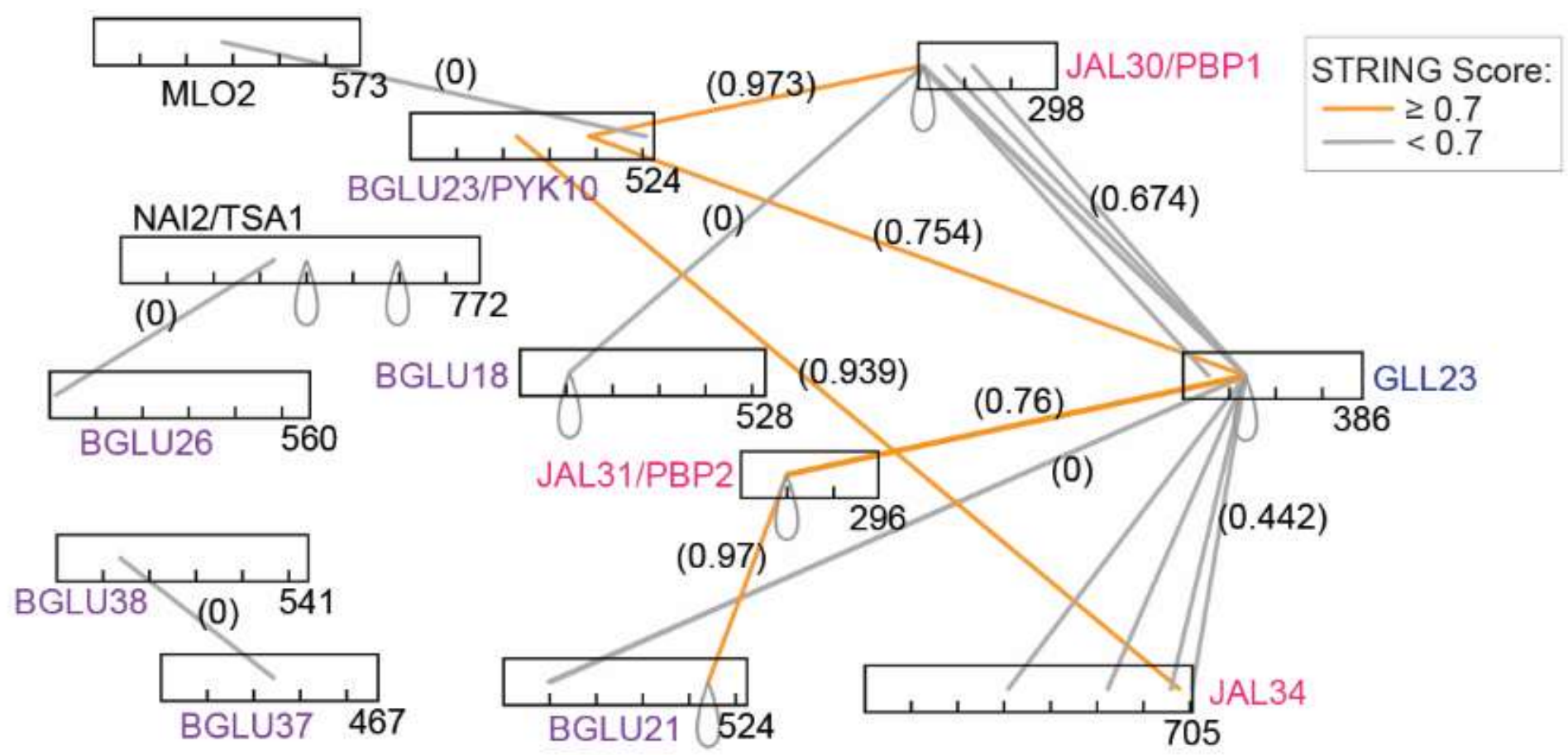

Supplementary Fig. S17: Interaction network of a specialized defense system detected by XL-MS.

XL-MS analysis revealed an extensive interaction network spanning multiple protein families involved in a specialized defense system. The network highlights extensive connectivity among BGLU family members, major constituents of ER bodies that encode β-glucosidases, together with jacalin-related lectin JAL/PBP proteins, which may function as molecular chaperones that facilitate proper BGLU polymerization, and GLL lipase-like proteins proteins. Additional interactions were detected with ER body protein NAI2, a master regulator of ER body formation, and MLO2, a homolog of the barley Mildew Resistance Locus O protein. The detection of both heteromeric and homomultimeric crosslinks suggests the formation of higher-order protein assemblies that may enable rapid activation of defense components upon cellular wounding. Although JAL/PBP proteins are normally localized in the cytoplasm, cellular wounding promotes their interaction with ER body proteins, including BGLUs and GLL23 proteins, leading to the formation of large protein aggregates and increased glucosidase activity. Lines are color-coded according to STRING confidence scores (orange, ≥0.7; grey, <0.7), with individual scores indicated in parentheses.

A

Data_Database	Software	Configured CPUs	Max Memory Usage (GB)	Search Time (hr)
ChloroF1_db9798	pLink3.2	24	38.6	0.77
ChloroF1_db9798	xiSearchv1.8.13+xiFDRv2.3.10	24	133.7	92.5
NuDark1F29_db14643	pLink3.2	24	44.8	1.1
NuDark1F29_db14643	xiSearchv1.8.13+xiFDRv2.3.10	24	133.3	88.6

B

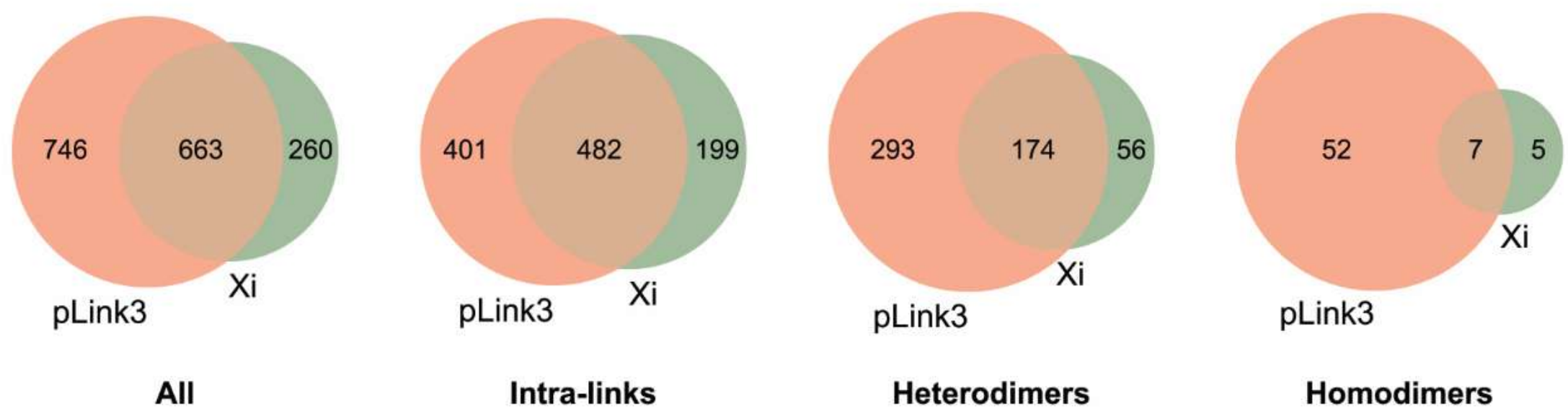

C

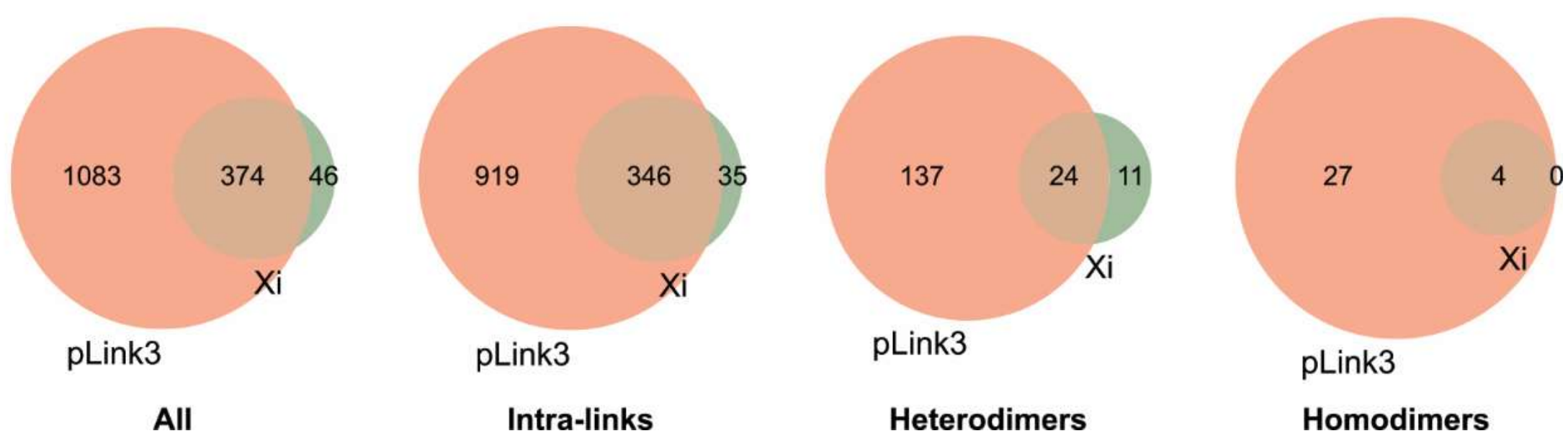

Supplementary Fig. S18. Comparison of data searches performed using pLink3 and xiSEARCH.

(**A**) Comparison of memory usage and search time between pLink3 and xiSEARCH using a single raw XL-MS data file. As xiSEARCH required longer processing times under our computational settings, whole-dataset comparisons were not performed. Instead, comparisons were conducted using two representative fractionation datasets: one chloroplast fraction and one nucleus fraction.

(**B–C**) Comparison of cross-linked peptide identifications obtained by pLink3 and xiSEARCH from single raw XL-MS datasets derived from chloroplast (B) and nucleus (C) fractions. A large proportion of cross-linked peptides were identified by both search engines, while each also detected unique cross-linked peptides. Overall, pLink3.2 identified a greater number of XL peptides.

Datasets

Dataset S1: Summary of cross-linked peptide, including intra- and inter- protein cross-links, PPI annotations based on STRING and FDR reports.

Dataset S2: SUBA5 annotation and Analysis of protein classification using PANTHER.

Dataset S3: mapping of intra-cross-links to known structures from PDB and predicted structure from AlphaFold, and simulation analysis comparison.

Dataset S4: Detected cross-links for ribosomal and histones PPIs, ribosome-related ubiquitination, H2B-TurboID.

Dataset S5: Names, accession numbers and description used in the figures and a glossary.

SI References

1. Xu S-L*, et al.* (2017) Proteomic analysis reveals O-GlcNAc modification on proteins with key regulatory functions in Arabidopsis. *Proc. Natl. Acad. Sci. U. S. A.* 114(8):E1536-E1543.
2. Shrestha R*, et al.* (2022) 15N Metabolic Labeling Quantification Workflow in Arabidopsis Using Protein Prospector. *Front. Plant Sci.* 13:832562.
3. Karunadasa SS, Grismer TS, Reyes AV, Zhai W, & Xu SL (2025) Detection and Quantification of Biotinylated Sites for TurboID-Based Proximity Labeling Mass Spectrometry in Arabidopsis. *Methods Mol Biol* 2953:127-141.
4. Mi H*, et al.* (2021) PANTHER version 16: a revised family classification, tree-based classification tool, enhancer regions and extensive API. *Nucleic Acids Res* 49(D1):D394-D403.
5. Zhang W*, et al.* (2023) ComMap: a software to perform large-scale structure-based mapping for cross-linking mass spectrometry. *Bioinformatics* 39(2).
6. Shrestha R*, et al.* (2025) Next-Generation Mapping of the ACINUS-Mediated Alternative Splicing Machinery and Its Regulation by O-glycosylation in Arabidopsis *BioRxiv* 10.1101/2025.01.04.631329.
7. Karunadasa SS, Grismer TS, Zhai W, Reyes AV, & Xu SL (2025) Detection and Quantification of Biotinylated Proteins for TurboID-Based Proximity Labeling Mass Spectrometry in Arabidopsis. *Methods Mol Biol* 2953:115-126.
